# Direct transcriptomic comparison of xenobiotic metabolism and toxicity pathway induction of airway epithelium models at an air–liquid interface generated from induced pluripotent stem cells and primary bronchial epithelial cells

**DOI:** 10.1007/s10565-022-09726-0

**Published:** 2022-05-31

**Authors:** Ivo Djidrovski, Maria Georgiou, Elena Tasinato, Martin O. Leonard, Jelle Van den Bor, Majlinda Lako, Lyle Armstrong

**Affiliations:** 1 The Biosphere, Newcells Biotech Ltd., Draymans way, Newcastle Helix, Newcastle upon Tyne, NE4 5BX UK; 2 Biosciences Institute, The International Centre for Life, Newcastle University, Central Parkway, Newcastle upon Tyne, NE1 3BZ UK; 3 Toxicology Department, Public Health England, Centre for Radiation, Chemical and Environmental Hazards, Harwell Campus, Chilton, OX11 0RQ UK; 4 Department of Medicinal Chemistry, Faculty of Science, Amsterdam Institute of Molecular and Life Sciences, Vrije Universiteit Amsterdam, Amsterdam, The Netherlands

**Keywords:** Induced pluripotent stem cells, Airway epithelium, Air–liquid interface, Toxicity assessment, Transcriptomics

## Abstract

**Abstract:**

The airway epithelium represents the main barrier between inhaled air and the tissues of the respiratory tract and is therefore an important point of contact with xenobiotic substances into the human body. Several studies have recently shown that in vitro models of the airway grown at an air–liquid interface (ALI) can be particularly useful to obtain mechanistic information about the toxicity of chemical compounds. However, such methods are not very amenable to high throughput since the primary cells cannot be expanded indefinitely in culture to obtain a sustainable number of cells. Induced pluripotent stem cells (iPSCs) have become a popular option in the recent years for modelling the airways of the lung, but despite progress in the field, such models have so far not been assessed for their ability to metabolise xenobiotic compounds and how they compare to the primary bronchial airway model (pBAE). Here, we report a comparative analysis by TempoSeq (oligo-directed sequencing) of an iPSC-derived airway model (iBAE) with a primary bronchial airway model (pBAE). The iBAE and pBAE were differentiated at an ALI and then evaluated in a 5-compound screen with exposure to a sub-lethal concentration of each compound for 24 h. We found that despite lower expression of xenobiotic metabolism genes, the iBAE similarly predicted the toxic pathways when compared to the pBAE model. Our results show that iPSC airway models at ALI show promise for inhalation toxicity assessments with further development.

**Graphical abstract:**

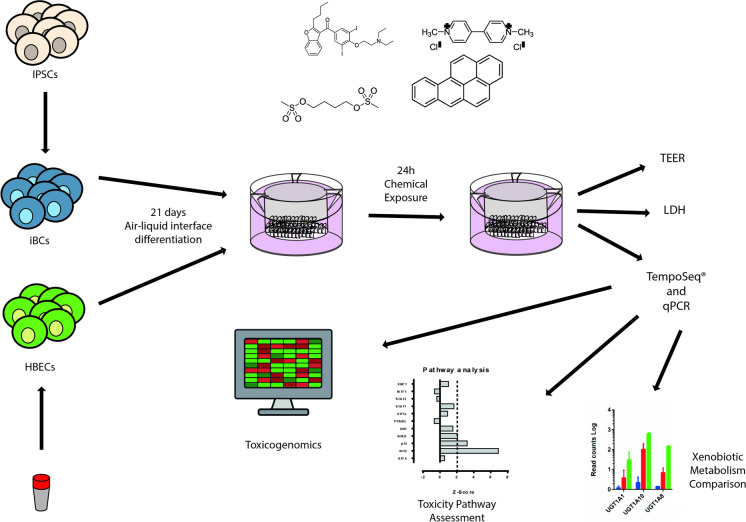

**Supplementary Information:**

The online version contains supplementary material available at 10.1007/s10565-022-09726-0.

## Introduction

The airway epithelium represents the primary barrier between inhaled air and the tissues of the respiratory tract and is therefore an important point of contact and/or entry of xenobiotic substances into the human body. Such substances may be subject to deliberate introduction required for delivery of therapeutic agents to treat respiratory diseases such as asthma and chronic obstructive pulmonary disease or accidental exposure such as entry of environmental pollutants, industrial chemicals, or household products. There is a need to assess the safety of materials entering the respiratory system. In the case of inhaled therapeutics, unacceptable levels of toxicity are a major obstacle to the development of new treatments, this being a major reason for attrition of drug candidates during therapy development which is reported to account for approximately 30% of inhaled drug project closures. A significant problem for the development of inhaled therapeutics is that toxicity is often detected only during pre-clinical in vivo testing in animal models which, although not a late stage of the development pipeline, will still have required considerable expenditure to progress to the point of failure. In view of this, methods to quantify or even predict the toxicity of xenobiotics are valuable tools since they have the potential to eliminate molecules at an earlier stage. Moreover, toxicity data from such models may also improve the design of downstream animal toxicity studies, thereby contributing to animal welfare in accord with the “3Rs” principles of replacement, reduction, or refinement. Although animal models are considered a “gold standard” for toxicity assessment, there is a drive to replace as many of these as possible since, quite apart from obvious ethical issues, the response of animals to drugs or other xenobiotics is not always a close parallel of the human response to the same materials. There are now global efforts underway to develop in vitro and in silico methods which can reliably predict the toxicity of chemicals, particularly by the EU with Directive 2010/63/ (Sykes et al. 2012; Olsson et al. 2016); however, there is also a pressing need to develop effective in vitro assays which replicate the response of human tissues as closely as possible.

A small number of air–liquid interface (ALI) in vitro cell-based models of the human airway are commercially available such as Mucilair™ (https://www.epithelix.com) and Epi-AirwayTM (https://www.mattek.com), and several of these have been evaluated both for assessment of toxicity of environmental toxins such as cigarette smoke, transport vehicle exhaust and industrial toxins and limited studies of inhaled drug toxicity (Sexton and Burube 2008; Huang et al. 2013). These types of models are constructed via culture of primary human airway epithelial cells at an ALI, whereupon they generate a pseudostratified airway epithelium composed of secretory, ciliated and basal cells (De Jong et al. 1994) that shows a degree of structural and transcriptomic similarity to the in vivo airway epithelium (Pezzulo et al. 2011; Aufderheide et al. 2015). Despite the utility of models, there are several drawbacks associated with the use of primary airway epithelial cells. The limited availability of cell donors is problematic, but the limited proliferation lifespan of these cells in vitro requires the repeated collection of donor tissues. In addition, genetic differences between the donors as well as lifestyle factors (smoking, dietary habits), medical history and donor age can directly impact the results of responses to toxic compounds. iPSC-derived tissue models avoid genetic variation between donors since the model can be constructed from a single iPSC line to ensure genomic uniformity. Moreover, iPSC offers the possibility to manufacture unlimited numbers of constructs from the same donor which is not possible using primary cells owing to the limited proliferation potential of the latter. Recently, several methods to generate iPSC-derived airway cells have been documented in the literature; however, none of the studies has examined the expression levels of genes involved in major toxicology pathways and xenobiotic metabolism. Lack of sufficient characterisation prevents the widespread application of iPSC models and their development for toxicological purposes. In view of this, we have developed an airway epithelial model generated from induced pluripotent stem cells (the iBAE model) at an ALI and directly compared this in parallel to a primary ALI model made from HBECs. We exposed the models to 5 chemicals (amiodarone, busulfan, cerium nanoparticles, paraquat dichloride and benzo(a)pyrene) at concentrations which do not result in cell death (referred to as sub-lethal concentration hereafter) to understand the induction of toxicity pathways and their effect on the expression of xenobiotic metabolism enzymes and transporters which has particular relevance in mechanistic toxicology. To measure the changes in gene expression, we used TempoSeq (Templated Oligo assay with Sequencing readout) (http://biospyder.com/) which is a commercial platform focused on mRNAs representing the major biological and toxicological networks. This comprises the s1500 gene set which identifies sentinel genes from known canonical pathways, which accurately predict pathway perturbations after chemical exposure (Mav et al. 2018). This is a cost-effective approach using RNA from cellular lysates removing the need to extract the RNA from the sample (House et al. 2017; Bushel et al. 2018). RNA-Seq has been employed for several studies of xenobiotic metabolism in the lung in vivo compared to in vitro models by looking at the expression of different xenobiotic metabolising enzymes, transporters and receptors (Boei et al. 2017; Courcot et al. 2012).

Transcriptomic analysis may also be used for the toxicological assessment of xenobiotic materials by quantitation of differentially expressed genes involved in specific gene regulatory networks or signal transduction mechanisms. Such genes may be grouped in so-called pathways of toxicity based upon the transcriptional response of genes in that pathway to specific xenobiotic materials. Several pathways have been described (https://www.wikipathways.org/). For example, XBP1 binds the sequence CCACG in a wide number of genes which respond to endoplasmic reticulum stress (Acosta-Alvear et al. 2007) and contributes to the unfolded protein response mechanism (Lee et al. 2003) of which ATF4 is also a part. The latter gene can also activate other components of the endoplasmic reticulum (ER) stress mechanism such as the C/EBP homologous protein transcription factor (CHOP), which engages downstream targets to initiate apoptosis in adverse conditions such as microbial infection (Hu et al. 2019). ATF4 has roles also in the control of translation initiation and stress-induced gene expression (Harding et al. 2000; B’chir et al. 2013) and is probably the best characterised effector in the signalling pathway referred to as the integrated stress response (Pakos-Zebrucka et al. 2016). Response to electrophilic and oxidising compounds is provided by the KEAP1-NRF2 antioxidant response element system (Kensler et al. 2007), which upregulates the expression of antioxidant defence genes (Prestera et al. 1995; Moinova and Mulcahy 1999), multidrug response transport proteins (Hayashi et al. 2003), and inhibitors of inflammation (Primiano et al. 1998) and initiates replacement of damaged proteins (Kwak et al. 2003). The aryl hydrocarbon receptor (AhR) is a transcription factor (part of the group of nuclear receptors) that responds to the presence of aromatic hydrocarbons by regulating genes involved in xenobiotic metabolism via binding to xenobiotic response elements (XREs) in their promoter regions. AHR targets genes of phase I and phase II metabolisms, such as cytochrome P450 1A1 (CYP1A1), cytochrome P450 1B1 (CYP1B1), NAD(P)H:quinone oxidoreductase I (NQO1) and aldehyde dehydrogenase 3 (ALHD3A1) (Gassmann et al. 2010). Other mechanisms involve the well-characterised p53 DNA damage response (Harris and Levine 2005), the NF-κB pathway (Lawrence 2009; Hayden 2008), regulation of energy metabolism by PPARG/ HIF1a and pathways resulting in activation of transcription factors such as STAT1. Understanding the induction of toxicity pathways can be used for mechanistic toxicology and is of particular importance for making safety assessments of drugs and chemicals.

The results of the transcriptomic analyses used in this study underline the similarities between human airway constructs generated by exposure of both iPSC-derived and ex vivo basal cells to an air–liquid interface. In particular, the expression of genes involved in the metabolic transformation of xenobiotic substances and the activation of toxic response pathways with sub-lethal chemical concentrations. Our study shows that iPSC models for the modelling of lung airways at ALI may be a useful tool to investigate the toxicity of diverse molecules towards the human airway epithelia which demonstrates the potential utility of the iBAE as a tool for drug development.

## Materials and methods

### Differentiation of iPSC to pseudostratified airway epithelium

Induced pluripotent stem cell line WT1 (SBAD2) (Buskin et al. 2018) was cultured at 37 ℃ + 5% CO_2_ on 6-well plates coated with Matrigel™ (BD, 354,230) in mTeSRTM1 (StemCell Technologies, 85,850) with daily media replacement. At 80% confluency, the cells were passaged with Versene EDTA 0.02% (Lonza, BE17-711E) for 5 min and transferred at a split ratio of 1:3 into fresh Matrigel-coated plates. The cells were passaged at least twice before initiating differentiation. Differentiation into airway basal cells involved transit through definitive endoderm and anterior foregut endoderm stages, as reported in our previous publication. To isolate basal airway-like cells, the day 14 differentiated cells were washed with PBS and enzymatically detached with trypsin for 5 min. The detached cells were centrifuged at 300 g and resuspended in BEGM medium (Lonza, CC-4175) supplemented with 10 µM of Y-27632. They were plated at a ratio of 1 well into 6 mitotically inactivated 3T3 cells. The medium was changed every other day until 90% confluency of basal cells was reached. The basal cells can be passaged at a ratio of 7000 cells/cm^2^ on irradiated 3T3s for at least 2 passages. After reaching 90%, confluency basal cells were harvested as a single-cell population by trypsinisation and then seeded at a density of 150,000 cells per well onto the apical face of 24 well plate cell culture inserts (ThinCerts™, Greiner bio-one, 662,610) with a transparent membrane (PET), with a pore diameter of 0.4 µm, pre-coated with Matrigel (1:100) and fibronectin (1:100) (Sigma Aldrich, F1141). The adherent cells were fed for three days apically and basolaterally with a BEGM medium until they formed a confluent monolayer. Once confluent, the apical medium was removed, and the cells were fed PneumaCult™ (Stem Cells Technologies, 05,001) supplemented with heparin, hydrocortisone and Pen/Strep from the basal chamber. The cells were cultured for 21 days with medium change every other day from the basal chamber.

### Differentiation of primary human airway basal cells to pseudostratified epithelium

Human bronchial epithelial cells (HBECs from upper airways, obtained from Dr Martin Leonard) from a single donor were thawed in a petri dish to allow colonies to grow in BEGM media. At 70% confluency, they were transferred into T75 flasks containing 3T3-J2 cells inactivated by X-irradiation and cultured with BEGM and rock inhibitor. Similarly, to iPSC-derived basal cells, iBCs at 90% confluency on mitotically inactivated 3T3 feeders were harvested as a single-cell population by trypsinisation and then seeded at a density of 150,000 cells per well onto the apical face of 24-well plate cell culture inserts (ThinCerts™, Greiner bio-one, 662,610) with a transparent membrane (PET), with a pore diameter of 0.4 µm, pre-coated with Matrigel (1:100) and fibronectin (1:100) (Sigma Aldrich, F1141). The adherent cells were fed for three days apically and basolaterally with a BEGM medium until they formed a confluent monolayer. Once confluent, the apical medium was removed, and the cells were fed PneumaCult™ (Stem Cells Technologies, 05,001) supplemented with heparin, hydrocortisone and Pen/Strep from the basal chamber. The cells were cultured for 21 days and fed every other day from the basal chamber. It is notable that the cell plating density exceeds that recommended by the manufacturers of PneumaCult™ since we optimised the density required to generate a monolayer of both iBCs and iPSC-derived basal cells within 48 h after plating. The recommended density of 40,000 cells did not generate monolayers within this timeframe.

### Characterisation of pseudostratified epithelia

The formation of an epithelial barrier was assessed by transepithelial resistance (TEER) as follows: Culture medium was replaced with fresh medium (500 μl/basolateral, 200 μl apical) equilibrated to ambient temperature (25 ℃). Resistance of the pseudostratified epithelium was measured using an EVOM2™ epithelial voltmeter and compared to the resistance of the transwell insert membrane in identical volumes of media but without the presence of cells. To obtain the TEER value in Ω.cm^2^, the blank (325Ω) is subtracted from the measurement and then multiplied by 0.334 (which is the area in cm^2^ of the membrane insert). A TEER value of above 300 Ω.cm^2^ is an indication of tight junction formation.

Airway constructs matured for 21 days were fixed directly on the membrane with 4% paraformaldehyde for 10 min at 3 ℃ and then washed with PBS (3 × 1.0 ml). The tissues were then removed together with the membrane, placed into moulds and embedded in optical coherence tomography (OCT) matrix (Cell Path, KMA-0100-00A). The moulds were placed at − 20 ℃ to solidify. Once solid, they were sectioned into 5–10 μm slices on slides using a cryostat. The sectioned membrane was removed with PBS washes, and the slides were then stained using antibodies listed in Table 1.

**Table 1:** Antibodies used for IHC analysis of iBAE and pBAE constructs

Antibody name	Dilution	Species	Reference	Supplier
CC10	1:100	Mouse	sc-365992	Santa Cruz
Synaptophysin	1:200	Rabbit	YE269	Abcam
ZO-1	1:300	Rabbit	61–7300	Invitrogen
Acetylated Tubulin	1:400	Mouse	T6793	Sigma Aldrich
α-Mouse-AlexaFluor-488	1:1000	Goat	A11001	Invitrogen
α-Rabbit-AlexaFluor-647	1:1000	Goat	A21245	Invitrogen

Expression of genes specific to the human airway epithelia with a specific focus on cytochrome P450 enzymes was performed using quantitative RT-PCR. Primers employed for this purpose are listed in Table 2.

**Table 2:** Sequences of primers used for quantification of human airway epithelial genes by Q-RT-PCR

Sequence name	Sequence
nGAPDH forward	GGTTTACATGTTCCAATATGATTCCA
nGAPDH reverse	ATGGGATTTCCATTGATGACAAG
MUC5AC forward	GCACCAACGACAGGAAGGATGAG
MUC5AC reverse	CACGTTCCAGAGCCGGACAT
FOXJ1 forward	GGCATAAGCGCAAACAGCCG
FOXJ1 reverse	TCGAAGATGGCCTCCCAGTCAAA
CC10 forward	TCATGGACACACCCTCCAGTTATGAG
CC10 reverse	TGAGCTTAATGATGCTTTCTCTGGGC
ASCL1 forward	CCCAAGCAAGTCAAGCGACA
ASCL1 reverse	AAGCCGCTGAAGTTGAGCC
CYP2F1 forward	ACCCTCCTTAACACCGTCCA
CYP2F1 reverse	ATGGCGGTGAGGTACAGAAAG
CYP2B6 forward	GCACTCCTCACAGGACTCTTG
CYP2B6 reverse	CCCAGGTGTACCGTGAAGAC
CYP2J2 forward	GAGCTTAGAGGAACGCATTCAG
CYP2J2 reverse	GAAATGAGGGTCAAAAGGCTGT
CYP2A6 forward	CGAGACCGTCAGCACCA
CYP2A6 reverse	GGATCACTGCCTCCATGT
CYP2S1 forward	CGCTACCACTGCTGGGAAACCT
CYP2S1 reverse	AATGGGCGTCCTTCTGTCCCC
CYP2E1 forward	CCTCCTGCTGGTGTCCATGT
CYP2E1 reverse	CTTGGGCTTGGGTCTTCCTGAGTGCT

### Analysis of exposure of pseudostratified airway epithelial constructs to xenobiotic materials

The following chemicals were added to iBAE and pBAE airway epithelial constructs: busulfan (500 µM), paraquat dichloride (100 µM) and amiodarone(15 μM). benzo(α)pyrene (500 μg/ml) and cerium nanoparticles (25 μg/ml) (10 μl aliquots) were added to the apical face of the constructs. Before adding the chemicals, the constructs were washed with PneumaCult™ (2 × 100 μl) to remove mucus build-up, so they were already moist before the application of the chemical treatment. When the 10 μl aliquots were deposited, they were added directly in the middle of the insert and then distributed across the whole monolayer by gently turning the plate. All treated constructs were incubated in contact with the xenobiotic substances (24 h, 37 ℃, 5% CO_2_), followed by quantification of TEER. Cytotoxicity was determined using the CyQUANT LDH Cytotoxicity Assay (Cat. Nos. C20300), which includes a positive control of exposure of the airway epithelial constructs to culture media plus 0.1% triton-X to ensure 100% cell death after 24 h exposure.

Airway epithelial constructs treated with xenobiotics as described above were subjected to transcriptomic analysis to compare the similarities of the iBAE model to the pBAE model and to quantify expression changes of genes involved in xenobiotic response and metabolism. Read count data obtained from TempoSeq™ were first normalised by scaling and subjected to quality control by plotting log-transformed bar charts of the normalised count data to detect outliers in the samples. The normalised read counts were analysed using DESeq2 (Love et al. 2014) to generate differentially expressed genes, calculating fold changes, *p*-values, base means and adjusted *p*-values. The default Wald statistics test was used for significance evaluation. The cut-off of 0.05 was set for adjusted *p*-values and > 2 or <  − 2 for log2-fold changes for the initial selection of the most significantly differentially expressed genes for the clustering of temporal profiles, development of the profile fitting procedure described below and identification of enriched pathways. The response of iBAE and pBAE to xenobiotics was subjected to principal component analysis as follows; the log-transformed and scaled TempoSeq count data was used for PCA (prcomp) using R 4.0. The PCA was visualised using the factoextra package.

## Results

### Airway epithelial basal cells generate pseudostratified airway epithelia at an air–liquid interface

**Fig. 1:**
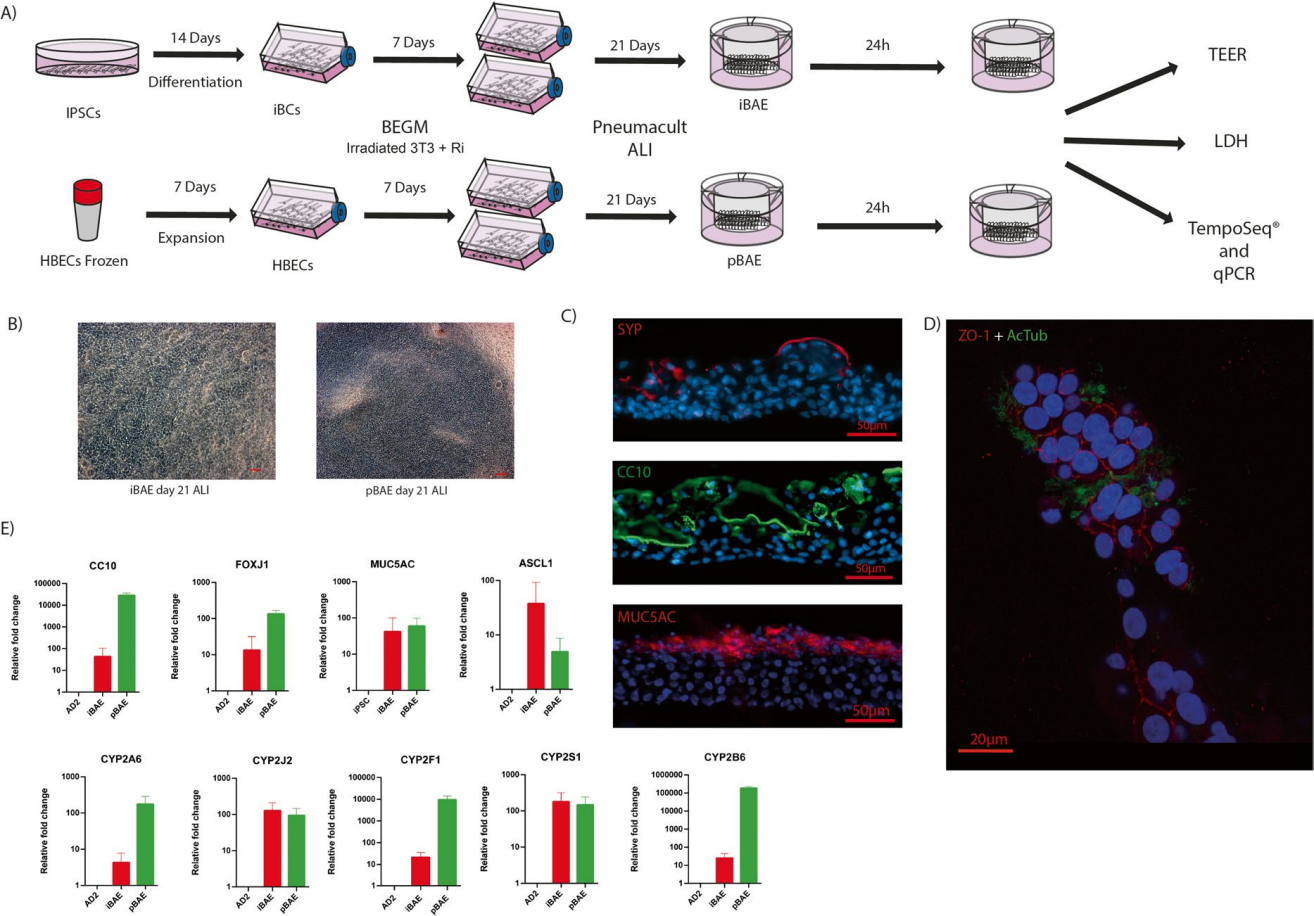
(**A**) Schematic protocol for generation of upper airway basal-like cells from iPSCs using the protocol detailed in the “Materials and methods” section, followed by differentiation at an ALI to produce pseudostratified airway epithelial constructs from both iPSC-derived and primary human bronchial epithelial cells (HBECs). (**B**) Brightfield images of the iPSC-derived (iBAE) and HBEC-derived (pBAE) constructs after 21 days differentiation at an air–liquid interface. Scale bars (red) in B represent 50 µm. Representative images from 6 experiments in both cell lines are shown. (**C**) Characterisation of the pseudostratified epithelia derived from iPSC-generated basal cells indicates the presence of pulmonary neuroendocrine cells: synaptophysin expression, club cell protein (CC10) expressing cells and Mucin 5AC expressing goblet cells. (**D**) The apical surface of the pseudostratified epithelium of iBAE constrcomprises ciliated epithelial cells capable of forming tight junction indicated by the presence of ZO-1 and cilia indicated by the presence of acetylated tubulin (AcTub). All scale bars for C and D as shown. Representative images from 6 experiments in both cell lines are shown. (**E**) Relative expression of marker genes of upper airway epithelium cell types in iBAE and pBAE constructs compared to undifferentiated WT1 iPSC line (*CC10* (club cells), *FOXJ1* (ciliated airway epithelial cells), *MUC5AC* (goblet cells) and *ASCL1* (pulmonary neuroendocrine cells)) measured by quantitative RT-PCR. Data are shown as mean + /SEM, *n* = 3 and relative expression of cytochrome p450 enzyme encoding genes in iBAE and pBAE constructs compared to undifferentiated WT1 iPSC line. Data are shown as mean + /SEM, *n* = 3

Airway constructs were generated according to the schematic diagram (Fig. 1A). Structures with mucous layers present on the apical surface were derived after this differentiation period (Fig. 1B), and sections of these structures indicate the presence of a pseudostratified epithelium (Fig. 1C). Differentiation of the basal cells into the other cell types present in the pseudostratified epithelium is indicated by the presence of club cell protein 10 (club cells, Fig. 1C) and expression of MUC5AC (goblet cells, Fig. 1C). The presence of putative pulmonary neuroendocrine cells is indicated by the expression of synaptophysin (Fig. 1C). In addition, the apical surface of the pseudostratified epithelium comprises ciliated epithelial cells capable of forming tight junctions indicated by the presence of ZO-1 and cilia indicated by the presence of acetylated tubulin (Fig. 1D). The ability to form tight junctions between the ciliated epithelial cells probably contributes to the TEER values in the range of 250–550 Ω/cm^2^ by day 60 of culture, indicating the establishment of an epithelial barrier. The expression of additional markers of airway phenotype was quantified by qRT-PCR, showing the expression of cytochrome P450 enzymes characteristic of the upper airway. In some instances (*CYP2J2*, *CYP2S1*, *MUC5AC*), expression levels were comparable between the iBAE and pBAE airway constructs, but for most genes (*FOXJ1*, *CC10*, *CYP2A6*, *CYP2F1*, *CYP2B6*), expression was lower in iBAE constructs (Fig. 1E). Expression of the pulmonary neuroendocrine cell marker *ASCL1* was higher.

Detectable expression of cytochrome P450 enzymes suggests that the iBAE airway epithelial construct might be capable of metabolism parallel to primary basal cell-derived constructs, albeit perhaps at lower levels of enzyme activity. In view of this, we proceeded to analyse the transcriptomic response of both types of the construct to xenobiotic compounds using TempoSeq analysis.

### iPSC-derived and ex vivo primary basal cell-derived airway constructs show comparable transcriptomes with respect to genes included in TempoSeq analysis

**Fig. 2:**
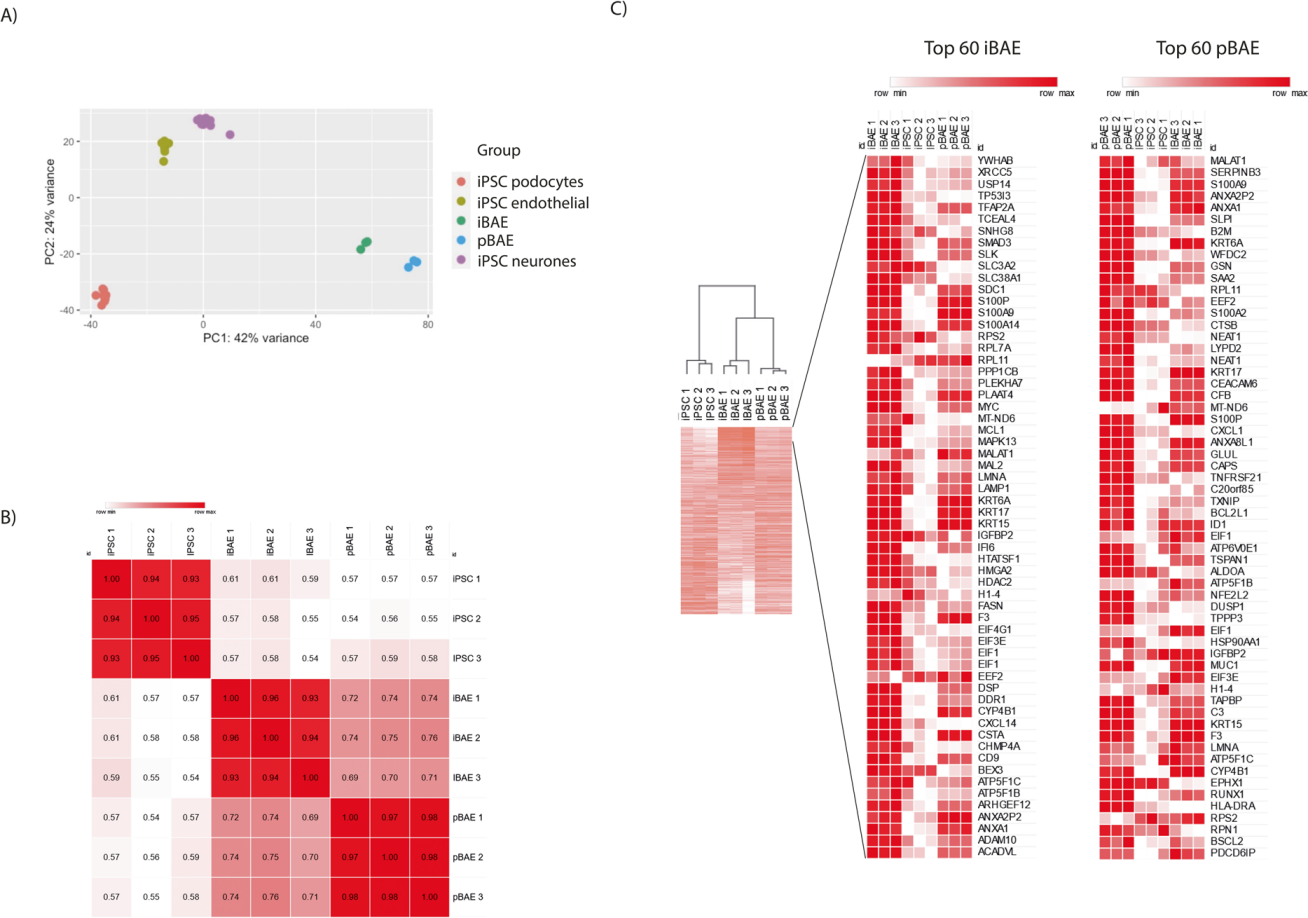
Temposeq™ transcriptomic analysis of the relative expression of genes represented on the Temposeq™ gene set between iBAE and pBAE models with comparison to undifferentiated iPSCs. (**A**) Principal component analysis shows that the expression of genes on the Temposeq™ data set clusters the iBAE and pBAE constructs together while distinguishing them from other iPSC-derived cell types generated by the IN3 consortium with lower variance between the airway constructs samples (*n* = 3) subjected to Temposeq™ analysis. (**B**) Pearson correlation indicated low variability between expression levels of Temposeq™ genes in iBAE and pBAE constructs, suggesting their similarity. Comparison of these models was extended to 60 highest expressed genes present in both models shown in the heatmap (**C**) of log2-fold changes generated using the software package *Pheatmap.* The heatmaps are arranged as follows: from left to right; three columns represent log2-fold changes for genes in triplicate for the iBAE model, three columns represent expression levels of the same genes in undifferentiated iPSC while three remaining columns represent three replicates of the pBAE model. Data presented are derived from triplicate repeats of Temposeq™ analysis using biological replicates of iBAE and pBAE constructs

Temposeq™ transcriptome data were analysed using the Deseq2 package. To obtain log2-fold change, TempoSeqR analysis software was used to generate the log2-fold differences. The similarity between samples and the heatmaps was generated by *Morpheus* (https://software.broadinstitute.org/morpheus). The normalised count comparison of xenobiotic metabolism was graphed using Prism GraphPad. Heatmaps of log2-fold changes were generated using the R package *Pheatmap.* Using principal component analysis (PCA), we observed that the transcriptomes of the iBAE constructs cluster together with those of the pBAE construct model and moreover have the lowest variance between each other when compared with PCA analysis of other iPSC-derived cell types produced by the IN3 consortium under which our current project was performed (Fig. 2A). We then compared the normalised values between the transcriptome data of undifferentiated iPSCs, iBAE and pBAE constructs and analysed the samples using Pearson correlation (Fig. 2B). This indicated low variability between samples (between 0.93 and 1) and similarity between the iBAE and pBAE models (*R* = 0.69¬0.76). A comparison of these models was extended to the 60 highest expressed genes present in both models; most of the top 60 highest expressed genes in the iBAE model (Fig. 2C) are also highly expressed in the pBAE model except for *Mt-ND6, CXCL14*, *TP53I3*, *EIF4G1* and *SLC3A2*, which are expressed either at lower levels or not at all in the pBAE model. Conversely, four genes (*NEAT1*, *HSP90AA1*, *EPHX1*, *TPPP3*) are expressed in the pBAE model that are not expressed by iBAE constructs. In both models, we can find highly expressed keratins (*KRT6A*, *KRT15*, *KRT17*) and annexins (*ANXA2P2*, *ANXA1*), which are characteristic of lung tissue. Mucin 1 (*MUC1*) was also found amongst the top 50 genes expressed in the primary basal cell model and is also highly expressed in the iBAE model. Other common genes that come up in both lists are the eukaryotic translation initiation factors (*EIF1*, *EIF3E*) and S100 calcium-binding protein P (*S100P*). The similarities observed in both models and expression of lung-specific genes suggest that iPSC differentiation was successful to obtain airway epithelium; however, there are still differences in terms of gene expression which could affect the metabolism and toxic response to xenobiotic chemicals.

### Expressions of nuclear receptors and phase I/II metabolic enzymes in iPSC-derived and ex vivo primary basal cell-derived airway constructs

**Fig. 3:**
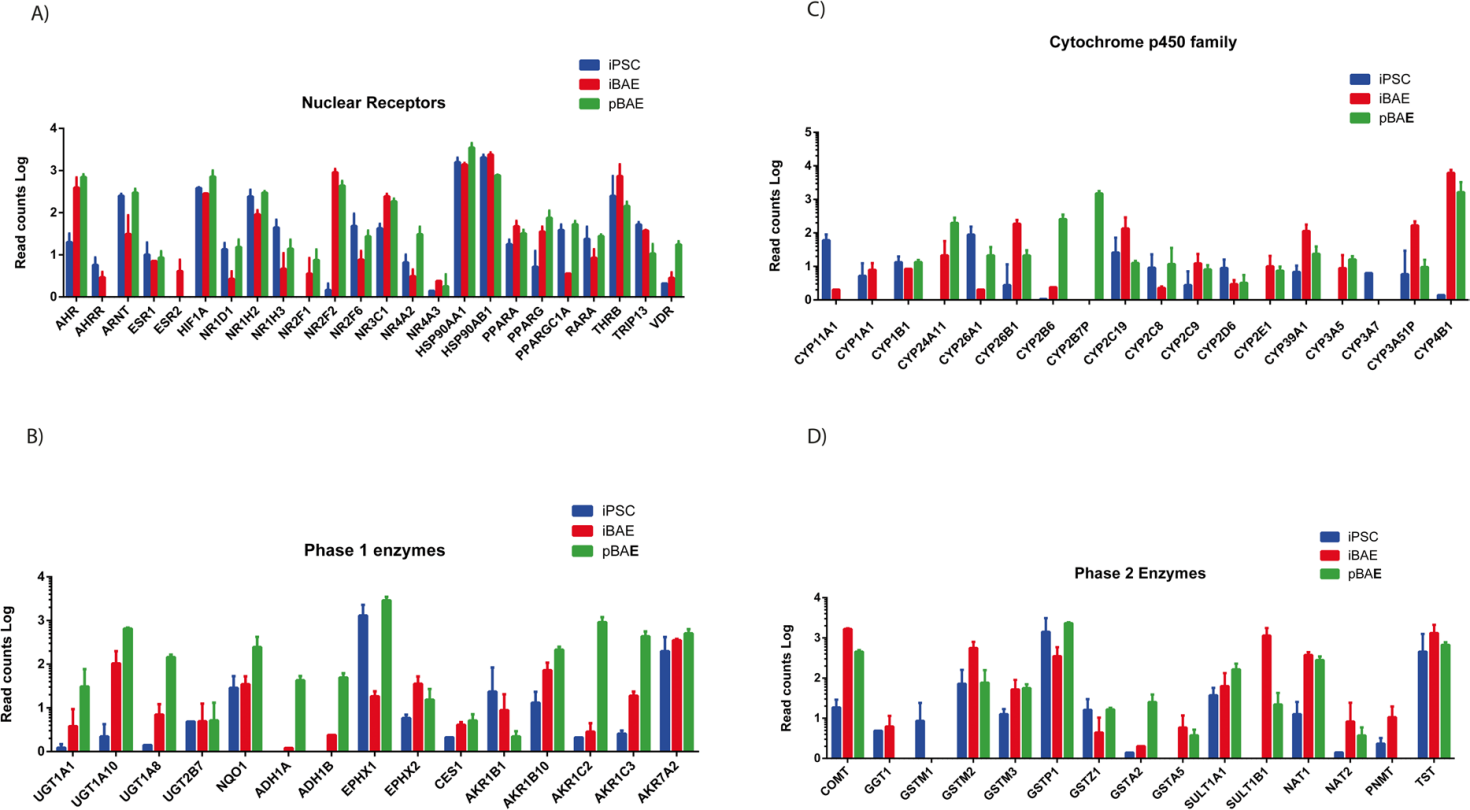
The iBAE and pBAE models show similar expression of (**A**) nuclear receptor encoding genes, (**B**) genes encoding enzymes involved in phase 1 metabolic transformation, (**C**) genes encoding cytochrome P450 family enzymes and (**D**) genes encoding enzymes involved in phase 2 metabolism (those which are present in the Temposeq™ data set). Data presented are derived from triplicate repeats of Temposeq™ analysis using biological replicates of iBAE and pBAE constructs

Organisms have developed a general strategy to protect themselves from possible toxic effects of xenobiotic molecules, which comprise two groups of molecular systems. The first line of defence involves nuclear receptors or xenosensors to detect the presence of potentially harmful materials and xenobiotic metabolising and transporter systems to break them down to substances that are more easily eliminated. We interrogated TempoSeq™ data to compare expression levels of key genes in these mechanisms. The aryl hydrocarbon receptor (encoded by *AHR*) which normally resides as an inactive complex with heat shock protein 9p chaperone protein and responds to the presence of planar aromatic hydrocarbons by translocation of the receptor protein to the nucleus, where it can activate the expression of xenobiotic metabolism genes (Göttlicher 1999). The translocation requires dimerization between the AHR protein and the gene product of *ARNT*, so it is encouraging that these two genes, in addition to the feedback repressor *AHRR*, are expressed in iBAE constructs (Fig. 3A). Interestingly, *AHRR* is not detected in the primary basal cell-derived constructs. Genes encoding the functionally similar oestrogen receptor *ESR1* are present in both pBAE and iBAE constructs, while ESR2 is detectable only in the latter. Several members of the nuclear receptor superfamily are expressed at similar levels in both models, while three members of the peroxisome proliferator-activated group of nuclear receptors (*PPARA, PPARG and PPARGC1A*), retinoic acid receptor alpha (*RARA*), thyroid hormone receptor β (*THRB*) and its interacting protein (*TRIP13*) and the vitamin D receptor gene (*VDR*) are also present at comparable levels. Together, these data suggest that the iBAE model may be a useful tool for quantifying the response of human airway epithelia to xenobiotic substances.

Phase I metabolism of xenobiotic substances aims to introduce or expose functional groups with the goal of increasing the polarity of the compound and broadly cover oxidation, reduction and hydrolysis reactions. The iBAE model expresses most of the enzymes involved in phase I metabolism that are present in the TempoSeq library (Fig. 3B), albeit these are mostly present at lower levels than in pBAE. The first group of genes presented in Fig. 3A are members of GDP-glucuronosyltransferase class (*UGT1A1, UGT1A10, UGT1A8, UGT2B7*). The aldo–keto reductases (*AKR1B1, AKR1B10, AKR1C2, AKR1C3, AKR7A2*) are also expressed, while several enzymes such as *UGT1A5,* the alcohol dehydrogenases (*ADH1A* and *ADH1B*) and *AKR1C2* were expressed at much higher levels in the pBAE model. The cytochrome P450 enzymes are important contributors to phase I metabolism due to their activity as monoxygenases capable of oxidising diverse xenobiotic substrates (Danielson 2002). *CYP1A1* is reported to be expressed in the human lung (Shimada et al. 1996; Shimada 2006) and to undergo significant induction by exposure to tobacco smoke (Kim et al. 2004), so it is interesting that it is only expressed by the iBAE model (Fig. 3C); however, the expression of other Cytochrome P450 genes correlates with published reports of their expression in the human lung (Carlson 2008). High expression of *CYP1A1* and *CYP4B1* relative to other members of the CYP class is reported in the literature [gene set – lung (maayanlab.cloud)], so it is useful to note that the iBAE model demonstrates greater expression levels than the pBAE model; however, *CYP2B7P* is the exception being detected only in the primary models. The iBAE model also shows a lower expression of *CYP2B6, CYP26A1* and *CYP2C8* than pBAE but also expresses *CYP2W1* which is one of the CYPs expressed in the bronchial mucosa in vivo (Courcot et al. 2012). That notwithstanding, the mRNA expression of phase I enzymes is comparable between the two models.

In summary, these data suggest that both iBAE and pBAE airway models should be capable of phase I/II metabolism of xenobiotic molecules.

### iPSC-derived and ex vivo primary basal cell-derived airway constructs show comparable expression of solute transporter protein genes

**Fig. 4:**
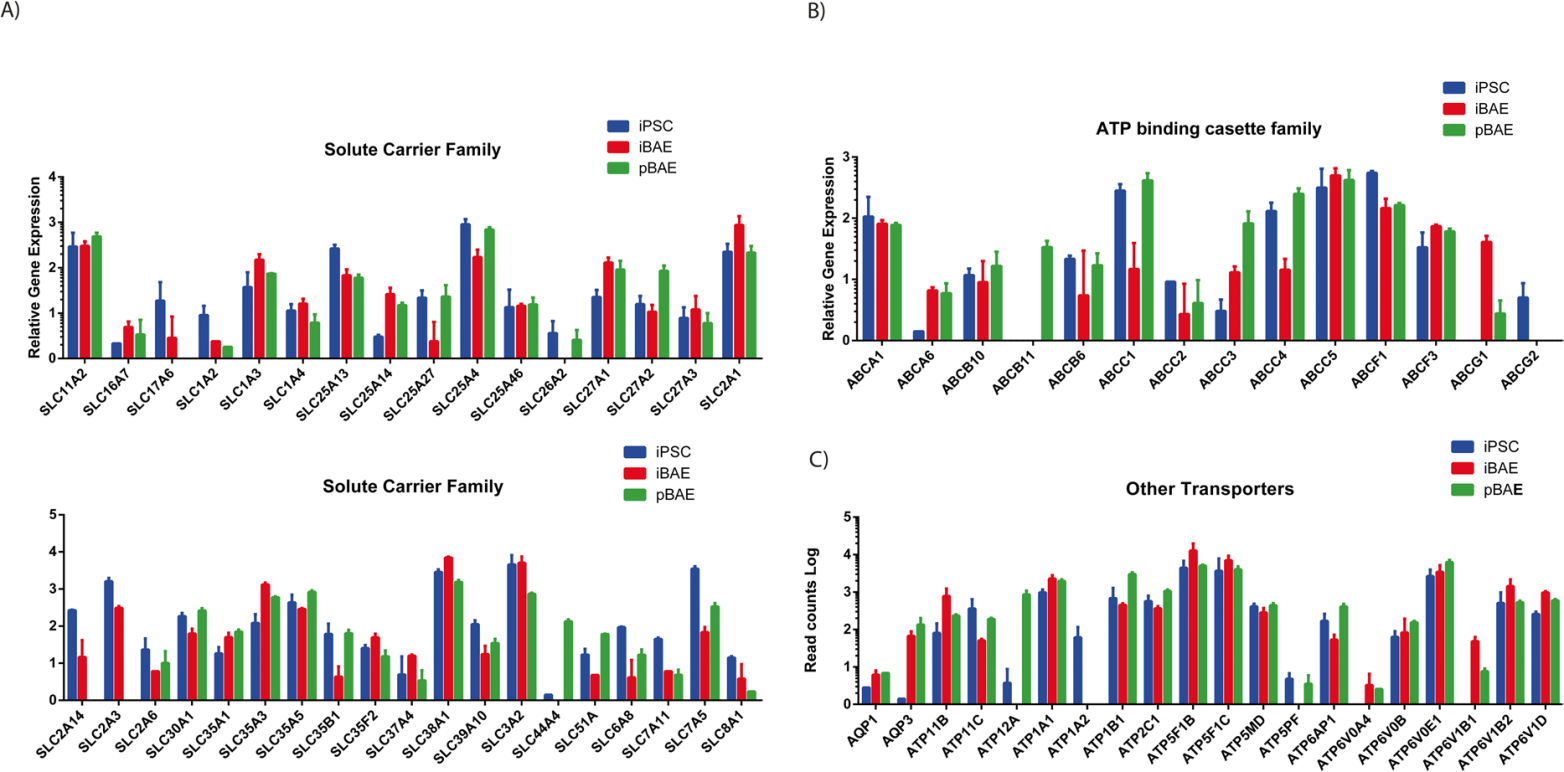
The iBAE and pBAE models show similar expression of **A** and **B.** Genes encoding solute carrier proteins **(C)** genes encoding ATP binding cassette family proteins and **(D)** other transporter proteins all classes of which are involved in the removal of xenobiotic molecules or their metabolites from cells of the upper airway epithelium. Data presented are derived from triplicate repeats of Temposeq™ analysis using biological replicates of iBAE and pBAE constructs

The solute carrier superfamily comprises over 400 transport proteins responsible for the movement of diverse substrates across membranes of the cell or its organelles (Hediger et al. 2004). Several transporter protein genes relevant for toxicological examination are expressed at comparable levels in both airway models (Fig. 4A) including neutral amino acid transporters (*SLC1A2, SLC1A3, SLC1A4*) (Kanai et al. 2013), glucose transporters (*SLC2A6, SLC2A1*) (Lizák et al. 2019), members of the mitochondrial carrier gene family (*SLC25A13, SLC25A14, SLC25A27, SLC25A4, SLC25A46*) (Gutiérrez-Aguilar and Baines 2013) and fatty acid transporters (*SLC27A1, SLC27A2, SLC27A3*) (Anderson and Stahl 2013) (Fig. 4A) with a few notable exceptions. The sulphate ion transporter (*SLC26A2*) (Heneghan et al. 2010) is absent in the iBAE airway constructs, as is choline transporter-like protein 4 (SLC44A4) (Nabokina et al. 2015). Conversely, two glucose transporter proteins (*SLC2A14* and *SLC2A3*) are absent from the pBAE models, although the impact of these discrepancies on the relative metabolic activities is not clear.

### Transcriptomic response of airway constructs to xenobiotic molecules

**Fig. 5:**
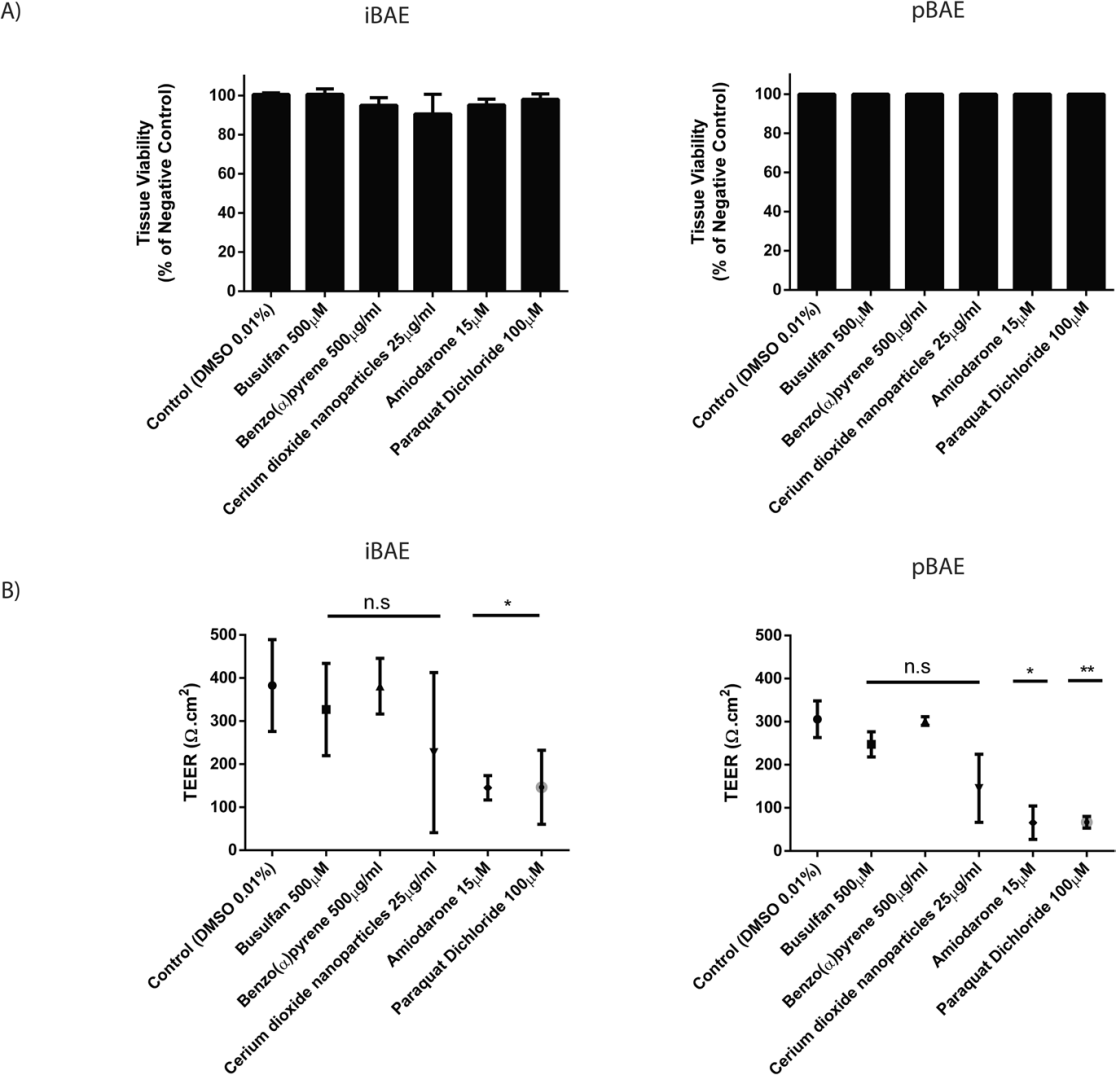
(**A**) Response to the iBAE and pBAE upper airway constructs to busulfan, benzo(a) pyrene, cerium dioxide nanoparticles, amiodarone and paraquat dichloride (24-h exposure) measured by the production of lactate dehydrogenase (LDH). Data are presented as mean + /SEM, *n* = 3, and expressed as percentage viability compared to negative control iBAE and pBAE constructs not exposed to either vehicle (DMSO) or test compounds. (**B**) Response of TEER to vehicle only (DMSO) and test compounds at the concentrations stated in the Fig. (24-h exposure). TEER measured in Ohms/cm^2^. Data are presented as mean + /SEM, *n* = 3

**Fig. 6:**
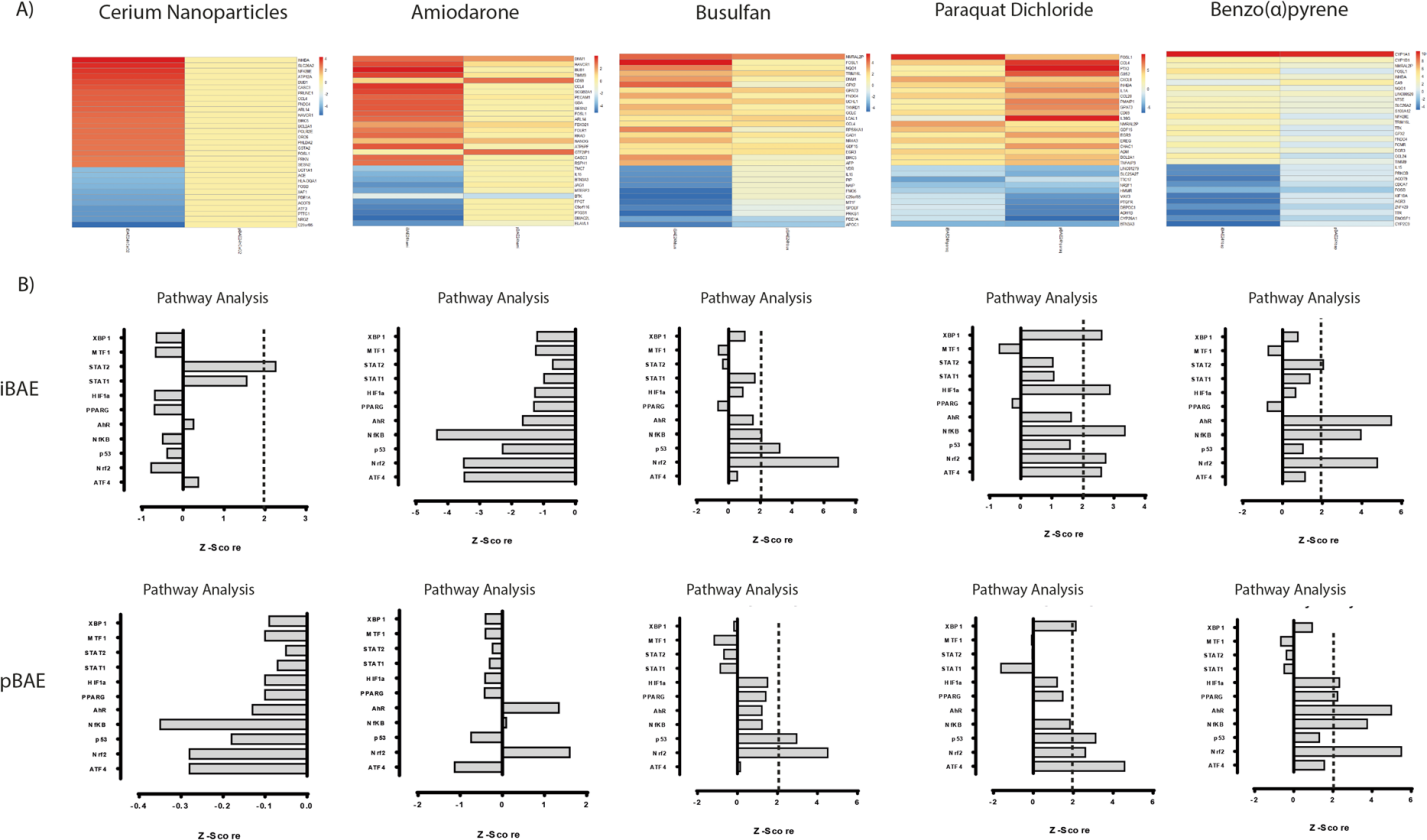
(**A**) Response of the iBAE and pBAE upper airway constructs to busulfan, benzo(a) pyrene, cerium dioxide nanoparticles, amiodarone and paraquat dichloride analysed by the number of genes from the Temposeq™ data set undergoing statistically significant log-fold change after 24-h exposure relative to untreated controls. (**B**) Analysis of log2-fold changes occurring in genes identified from by Temposeq™ analysis that are participants in the major toxicity-associated pathways (ATF4, NRF2, NFkB, AhR, PPARG, HIF1a, STAT1/2, MTF1 and XBP1) by quantification of *Z*-score (see Supplementary Table 1 for the method of *Z*-score calculation). Data presented are derived from triplicate repeats of Temposeq™ analysis using biological replicates of iBAE and pBAE constructs

iPSC and primary basal cell-derived models were exposed to five xenobiotic substances with known toxicity against lung/airway tissues at sub-lethal concentrations over 24 h. Cytotoxicity was assessed by quantifying TEER and LDH, although we did not find any significant increase in the LDH activity relative to the untreated control, suggesting that the concentrations of chemicals used do not kill cells in the airway constructs. Measurement of TEER before and after treatment indicated that only amiodarone and paraquat caused a significant reduction in TEER, suggesting a breakdown of the epithelial barrier (Fig. 5A, B). Data obtained from TempoSeq analysis of the transcriptomes of xenobiotic treated cells were processed using the Deseq2 package to generate lists of statistically significant log-fold change lists of genes affected by chemical treatment relative to the untreated control (Love et al. 2014) (Fig. 6A) and subjected to principal component analyses to quantify the similarity of response to the applied compounds of the iBAE and pBAE models (Supplementary Fig. S1A–E). In performing this analysis, we aimed to show the correlation between the untreated iBAE and pBAE models and between the individual models after treatment with the xenobiotic compounds. The analyses in Supplementary Fig. S1 show that principal components 1 and 2 demonstrate the variance between the iBAE and pBAE models before and after xenobiotic treatment, while principal components 3 and 4 account for the variance within the models. These data indicate significant differences between the iBAE and pBAE models; however, given the degree of similarity in expression of genes involved in phase 1 and 2 metabolisms, we considered the possibility that variance may have arisen from the genetic background of the patient-derived cells used to generate the pBAE model and the iPSC used to generate the iBAE model.

We then selected the top 20 upregulated and 10 top downregulated genes with the highest sum of log2-fold changes with significantly low *p*-value adjusted (*p* < 0.05). We applied gene ontology analysis to the top 20 genes (up and downregulated); however, the limitation of gene ontology analysis is that it is not specific to toxicological processes, so we manually annotated the genes identified by TempoSeq that are participants in the major toxicity-associated pathways (ATF4 **(**HU et al. 2019**; **Lee et al. 2003**)**, Nrf2 (B’Chir et al. 2013), p53 (Kwak et al. 2003), NFkB (Gassmann et al. 2010; Harris and Levine 2005), Ahr (Guerrina et al. 2018), PPARG (Michalik et al. 2006), HIF1a (Masoud and Li 2015), STAT1/2 (Xu et al. 2012), MTF1 (Laity and Andrews 2007) and XBP1 (Boei et al. 2017**)** (see Supplementary Table 1 for list of genes assigned to each toxicity pathway). These data were analysed using PathVisio3 **(**Kutmon et al. 2015**)** to analyse the log2-fold changes of genes induced by the treatment of the chemicals and calculate a Z-score based on the annotation of genes. The pathways are ranked based on their *Z*-score, in which a positive *Z*-score (*Z* > 1.96) indicates a pathway with more genes meeting the criterion than expected based on the complete dataset. A negative *Z*-score (*Z* < 1.96) indicates that less genes meet the criterion than expected. An examination of *Z*-score data suggests that iBAE airway models are marginally more susceptible to toxicological pathway activation than pBAE models (Fig. 6B). After the application of cerium nanoparticles, the only pathway that shows statistically significant activation in the iBAE model is STAT2, although 87 genes show differential expression between test and control samples. Amongst the top 20 upregulated genes, we can see genes involved in several different processes such as apoptotic response (*BIRC5, PHLA2),* cell cycle arrest and differentiation (INHBA), NFkB inhibition and ciliogenesis (*SLC26A*) (Supplementary Table 1).

Amiodarone is a potassium channel blocking agent indicated for life-threatening supraventricular and ventricular arrythmias (Baritussio et al. 2001), but the use of this drug is associated with significant toxic effects against lung tissue, primarily the development of pulmonary fibrosis. As an amphiphilic cationic molecule, amiodarone inhibits lysosomal phospholipases, leading to disruption of the lysosome membranes (Halliwell 1997) and release of reactive oxygen species which may lead to stress pathway activation and lung epithelial cell apoptosis. Surprisingly, our TempoSeqTM analysis of amiodarone exposure did not indicate significant activation of toxicological pathways in either the iBAE or pBAE models. Conversely, significant downregulation of pathways involving *NF-kB*, *p53, NRF2* and *ATF4* is observed in the iBAE model, meaning that other genes outside the pathways are strongly affected. Differentially expressed genes were detected for other GO classes, *HAVCR1* (cilia function), *BUB1* (apoptosis), *CCL4* and *PECAM1* (cell adhesion) (Supplementary Table 1).

Busulfan is an alkylating anti-neoplastic agent used to treat chronic myeloid leukaemia (Chen et al. 2013) which can cause interstitial lung fibrosis in humans (Oliner et al. 1961). In both models, Nrf2 was activated (with *Z*-score 6.9 in iBAE and 4.5 in the pBAE) and p53 (with *Z*-score 3.2 in iBAE and 2.97 in the pBAE), while NFkB was only affected in the iBAE (*Z*-score = 2.07). The most upregulated genes in the iBAE are *NMRALP2* (redox sensor), *FOSL1* (involved in cilia), *NQO1* (NADP(H)dehydrogenase involved in Nrf2), *DNM1, GPX2, CCL4* and *BRICS.* In the pBAE, it is *NMRALP2*, *TRIM16L*, *GPHT* and *UHCL1* (Supplementary Table 1). Activation of *p53* and *NRF2* is consistent with a response to DNA damage induced by an alkylating agent such as busulfan.

Paraquat (*N*,*N*′-dimethyl-4,4′-bipyridinium dichloride) is a redox-active heterocyclic compound which functions as an electron transport chain blocking agent. Paraquat has been widely employed to trigger oxidative stress given its unique potency to generate superoxide anion in nearly all experimental systems ranging from isolated mitochondria and mammalian cells (Blanco-Ayala et al. 2014) and as such its mechanism of toxicity is thought to arise by exceeding the capacity of cells to eliminate reactive oxygen species. Several pathways are downstream targets of this imbalance such as NF-KB (Gonzalez-Polo et al. 2004) and UPR (Mottis et al. 2014), so it is interesting to note the activation of *XBP1, HIF1α, NF-KB, NRF2* and *ATF4* in the iBAE model following paraquat exposure (Fig. 6B). It is noteworthy that *NF-KB* and *HIF1α* activation in the pBAE airway model fall short of statistical significance of their *Z*-scores, while the p53 DNA damage response correlates with reported increases of double-strand break formation following paraquat exposure in cultured mouse lymphoblasts, albeit at a 10 × greater concentration than used in our study (Ross et al. 1979). Differentially expressed genes in the iBAE and pBAE models following paraquat exposure are shown (Supplementary Table 1).

The polycyclic aromatic hydrocarbon, benzo(a) pyrene, has very similar effects on both models with activation of the NF-KB, NRF2 and aryl hydrocarbon response pathways. The iBAE model shows activation of STAT2 that is on the boundary of statistical significance (*Z*-score = 2.06), which was not seen in the pBAE model, while the latter also shows exclusive but modest activation of HIF1α. It is surprising that activation of the DNA damage response, p53, was not observed in either model given the reported ability of benzo(a)pyrene to induce double-strand DNA breaks (Wang et al. 2005); however, increased activation of AHR, NRF2 and AT4 are consistent with reported toxicity mechanisms of this molecule (Jin et al. 2021). Differentially expressed genes in the iBAE and pBAE models following benzo(a) pyrene are shown (Supplementary Table 1).

## Discussion

Tissue models derived from iPSC are potentially useful in the toxicological assessment of xenobiotic substances. They not only offer unlimited supplies of cells but can also be genetically edited to target specific transporters or enzymes and delineate toxicological pathways. Moreover, they offer the possibility to test the response of cells generated from any individual which may be useful for understanding drug toxicity and efficacy in the pre-clinical phase of drug development. ALI in vitro models of human airways for toxicity assessment have been developed using airway epithelial cells harvested from patients, so the objective of this study was to investigate the potential of iPSC-derived in vitro models of the human airway for quantification of toxicological responses. ALI constructs similar to the human airway epithelium were generated using airway basal cells generated from iPSC-derived basal airway cells (iBAE model) and from primary basal cells obtained from a human donor (pBAE model). Both models demonstrate similar morphology and the presence of expected cell type; however, a more detailed characterisation of their transcriptomes was performed using TempoSeq analysis as required by the consortium from which the funding to support this study was derived. While this method is useful for determining toxicological responses of a variety of cell types, it is less effective as a tool to confirm differentiation towards an airway phenotype since key lung markers genes are not represented in the TempoSeq gene set. The absence of lung phenotypic markers does not impact upon the effectiveness of temposeq as a tool for analysing toxicological responses. We characterised our constructs using qPCR, which suggest that the iBAE model is perhaps less mature than the pBAE since there is a lower expression of goblet (*MUC5AC*), club (*CC10*) and ciliated marker (*FOXJ1*) while having a higher expression of neuroendocrine marker (*ASCL1*). That said, we have a high degree of confidence that our iPSC differentiation protocol generates airway epithelia since (1) it has similarities to existing published protocols and (2) we employ an intermediate step to expand cells of an airway basal cell phenotype (Djidrovski et al. 2021); thus, there is less likely that the protocol will generate cells with phenotypes that are similar to those found in the alveoli.

Markers of epithelial basal cells (*KRT6A, KRT17)* and airway epithelium (*ANNEXIN1, ANNEXIN2, MUC1, CYP4B)* that are present on the TempoSeq gene set are amongst the highest expressed genes detected by this analysis; therefore, we are confident of the iBAE model’s similarity to airway epithelium. Moreover, the top 50 genes detected by TempoSeq analysis of the iBAE and pBAE models are expressed in common, indicating a high degree of similarity between the iPSC and primary basal cell-derived models. Since the iBAE and pBAE models are from different donors, we compared the temposeq datasets from both using principal component analysis which indicates a reasonable degree of correlation between both models. This is further underlined by the similar expression levels of genes involved in the detection, metabolism and transport of xenobiotic substances, although there are several noteworthy differences. The expression of nuclear receptor genes in iBAE mostly parallels those of pBAE, except for *ESR1* and *AHRR*; however, two genes encoding enzymes which contribute to phase 1 metabolism are expressed only by the iBAE model (*CYP11A1, CYP1A1*) compared to the pBAE model in which exclusive expression of only *CYP2B7p* is observed. The consequences of these differences are unclear, but it is encouraging that the iBAE model expresses cytochrome P450 genes reported to be preferentially expressed in human lung (*CYP1A1*, *CYP1B1, CYP4B1, CYP2E1 and CYP3A5*) (Zhang et al. 2006) at comparable or superior levels to pBAE. Of these genes, *CYP1A1* and *CYP1B1* show significant induction to xenobiotic materials such as exposure to cigarette smoke (Willey et al. 1997), whereas *CYP2B7P* does not despite its high expression level in lung tissue. All other phase 1 metabolism genes show common expression, albeit some are present at lower levels in the iBAE model.

The focus of phase 2 metabolism is the solubilisation of xenobiotic substances or their metabolic products to facilitate elimination. Both the iBAE and pBAE models express a range of phase 2 metabolism genes represented on the TempoSeq gene set, although again, there are notable exceptions. None of the genes in this class are exclusive to the pBAE model, but γ-glutamyl transferase and phenylethanolamine N-methyltransferase (*GGT1* and *PNMT*, respectively) are only expressed in iBAE. Glutathione is an essential factor in protecting the pulmonary system from toxic insults (Potdar et al. 1997), and γ-glutamyl transferase expression can be detected in adult type II alveolar epithelial cells and Clara cells of the upper airway (Jean et al. 2003). Both cell types need extensive antioxidant defences and require glutathione as a substrate for xenobiotic metabolism with a particular focus on the upper airway since it is a primary site for the deposition of inhaled particulate matter. Phenylethanolamine N-methyltransferase is the terminal enzyme of the catecholamine biosynthesis pathway and normally methylates norepinephrine to convert this into the active form epinephrine (Ji et al. 2005). This enzyme has been reported to methylate the monoamine oxidase inhibitor phenelzine (Yu et al. 1991); however, methylation is generally a minor pathway of xenobiotic transformation (Jancova and Siller 2012), so the expression of *PNMT* is an interesting observation but may be of lower significance than *GGT1*.

The absence of glutathione-S-transferase μ1 (*GSTM1*) in both models is interesting since lack of GTSM expression has been linked to an increased risk of developing adverse health effects following exposure to pro-oxidant air pollutants such as diesel exhaust and cigarette smoke (Peden 2005; Romieu et al. 2004). *GSTM1* is regulated by NRF2 which is expressed by both models, so its absence is difficult to explain. For the pBAE model, this could be cell line-dependent and may correlate with the occurrence of the so-called *GSTM1* null phenotype reported in up to 62% of individuals of European descent (Geisler and Olshan 2001); however, the undifferentiated iPSC line used to generate the iBAE model clearly expresses GSTM1, so this explanation is unlikely for the iPSC-derived airway construct. Other glutathione-S-transferases (*GSTM2 & GTSM3*) are expressed at comparable levels by both models.

The TempoSeq gene set contains a broad range of solute channel transporter proteins, and the majority of these show comparable, although not precisely equal, expression levels between iBAE and pBAE. Notable exceptions include members of the glutamate transporter channel class (*SLC17A6*), sulphate transporter (*SLC26A2*), glucose transporters 14 and 3 (*SLC2A14, SLC2A3*) and choline transporter-like protein 4 (*SLC44A4*). The impact of these proteins on the capability of either model to transport xenobiotic substances and their metabolites across the membranes of the cells that comprise the iBAE and pBAE models is not clear.

The primary focus of this study was to characterise the transcriptional responses of the two models to xenobiotic exposure and to gain insights into the induction of toxicity pathways at sub-lethal concentrations of the drugs as described. We chose to restrict our study to the iBAE and pBAE models since the latter already has commercial applications for toxicity assessment; therefore, our aim was to assess the compatibility of pBAE with the more readily manufactured iBAE model. That notwithstanding, comparison of both models with ex vivo derived human airway tissue is a valuable undertaking that can be developed in future work to develop previously published studies that quantified gene expression in airway tissue and bronchial epithelial cells (Courcot et al. 2012). Analysis of the metabolites generated by each model would be the next step in understanding the toxicological response of each model in more detail, but this was outside the scope of the current study. The TempoSeq data obtained indicate some similarities between iBAE and pBAE that may allow the use of the iBAE model as a tool for toxicological assessment. With that in mind, we exposed both models to 5 compounds with known lung toxicities and analysed transcriptomic changes using TempoSeq. Molecules such as paraquat, busulfan and benzo(a) pyrene activate pathways such as the aryl hydrocarbon response, p53, nRF2 and ATF4 in line with expectations, while amiodarone and cerium oxide particles show repression of many pathways rather than statistically significant activation. This may be related to the known effects of the substances. Amiodarone is reported to cause pulmonary fibrosis upon chronic exposure to the drug, but iBAE and pBAE models cannot address this since they only comprise a pseudostratified airway epithelium and lack the stromal and vascular components that would normally contribute to fibrotic development. This latter observation highlights a key limitation of this study in that we can only use the models in their current forms to assess the toxic response of the airway epithelium, and a more comprehensive study would require not only a greater range of substances over a range of reported toxicities directed against the airway but also the inclusion of greater structural complexity to fully replicate the cellular architecture of the airway. In addition, the use of a “bulk” RNA sequencing approach on a complex mixture of cell types such as the iBAE and pBAE models does not quantify the toxicological responses of individual cell types; therefore, single-cell RNA sequencing may be required in future studies to obtain these data. To conclude, this study shows that there are transcriptomic similarities of genes involved in xenobiotic metabolism between iPSC and primary ALI airway models. We show that iPSC models can be very useful for studying toxicity and are in need of future studies to address functional metabolism by looking at metabolites and transporter functions.

## Supplementary Information

Supplementary Figure 1 Principal component analyses of response of the iBAE and pBAE models to (A) amiodarone, (B) busulfan, (C) benzo-a-pyrene, (D) cerium nanoparticles and (E) paraquat. For each treatment the individual samples (small dots) and their average (large dot) of the first four principal components are visualized. These analyses show that principal components 1 and 2 demonstrate the variance between the iBAE and pBAE models before and after xenobiotic treatment while principal components 3 and 4 account for the variance within the models. (PNG 216 kb)

High resolution image (TIF 463 kb)

ESM 2 (ODT 99 kb)

## Data Availability

The datasets supporting the conclusions of this article are included within the article and its supplementary files.
