# Supplementary figures and images for: Direct transcriptomic comparison of xenobiotic metabolism and toxicity pathway induction of airway epithelium models at an air–liquid interface generated from induced pluripotent stem cells and primary bronchial epithelial cells
Source: Cell Biol Toxicol. 2022 May 31;39(1):1–18. doi: 10.1007/s10565-022-09726-0 (PMC10042770; doi:10.1007/s10565-022-09726-0)

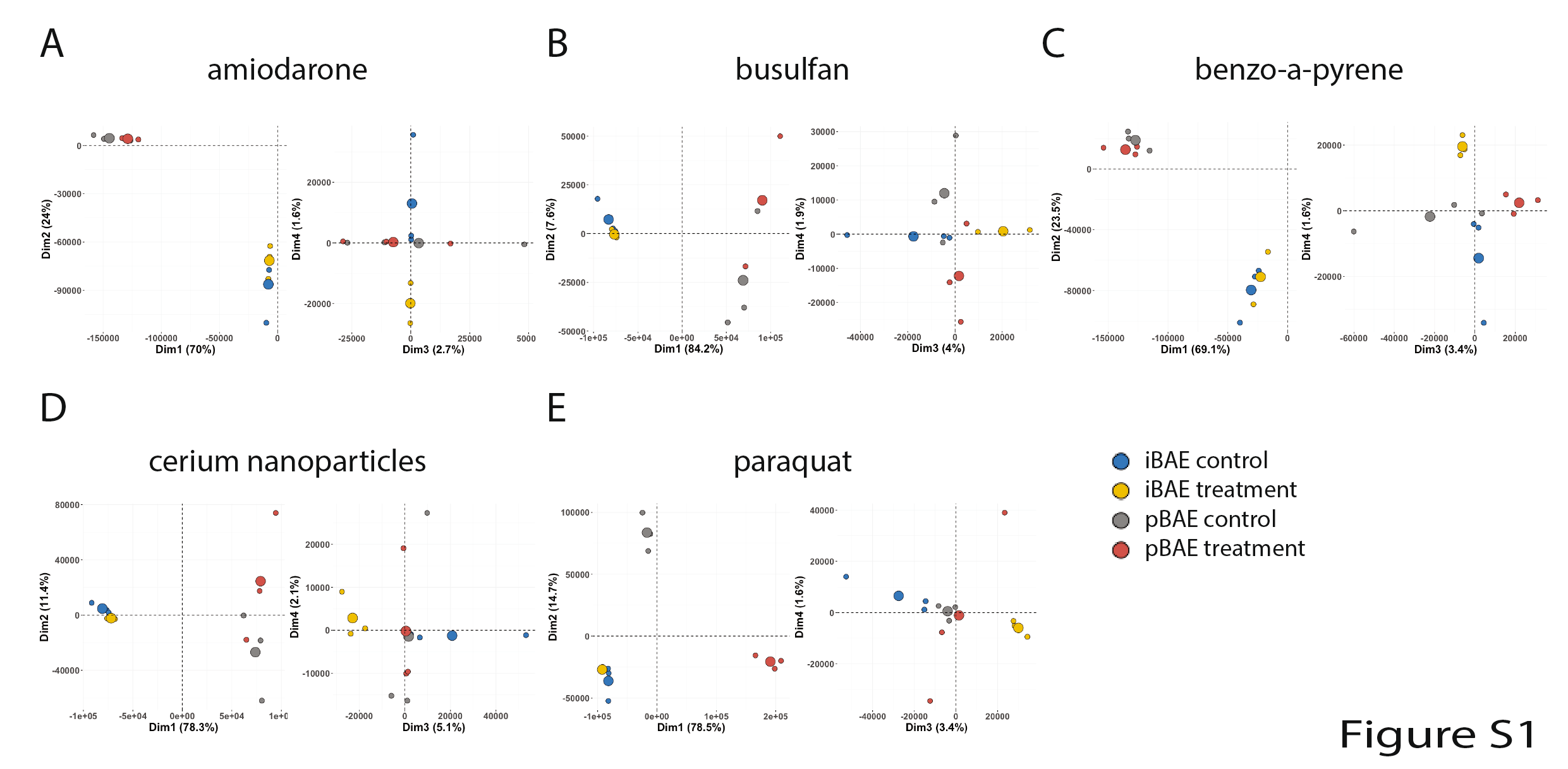

Supplement: Supplementary file 1 — Principal component analyses of response of the iBAE and pBAE models to (A) amiodarone, (B) busulfan, (C) benzo-a-pyrene, (D) cerium nanoparticles and (E) paraquat. For each treatment the individual samples (small dots) and their average (large dot) of the first four principal components are visualized. These analyses show that principal components 1 and 2 demonstrate the variance between the iBAE and pBAE models before and after xenobiotic treatment while principal components 3 and 4 account for the variance within the models. (PNG 216 kb) [file 10565_2022_9726_Fig7_ESM.png]

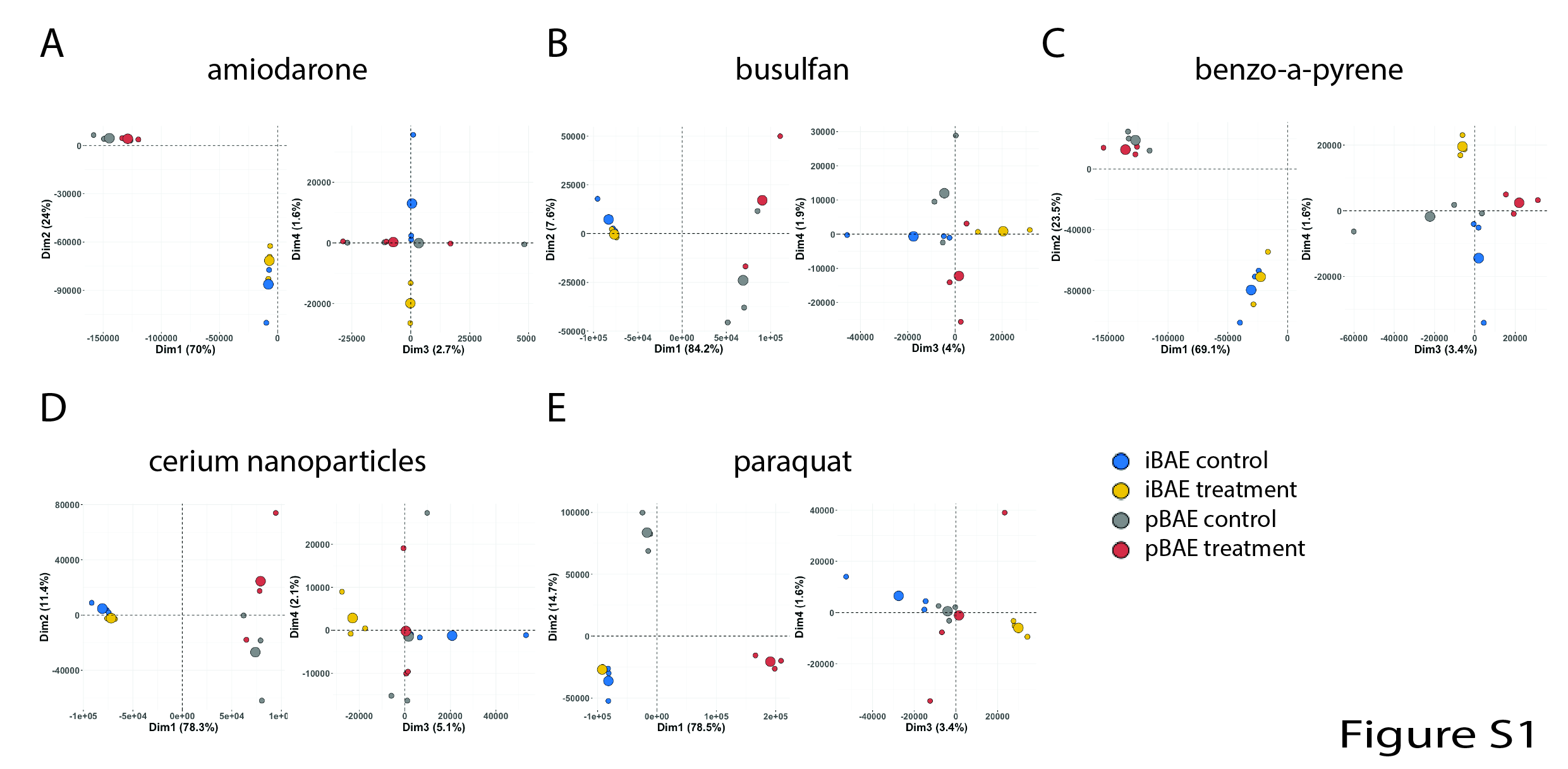

Supplement: Supplementary file 2 — High resolution image (TIF 463 kb) [file 10565_2022_9726_MOESM1_ESM.tif]
