# Supplementary material for: Direct transcriptomic comparison of xenobiotic metabolism and toxicity pathway induction of airway epithelium models at an air–liquid interface generated from induced pluripotent stem cells and primary bronchial epithelial cells
Source: Cell Biol Toxicol. 2022 May 31;39(1):1–18. doi: 10.1007/s10565-022-09726-0 (PMC10042770; doi:10.1007/s10565-022-09726-0)
Supplement: Supplementary file 2 [file 10565_2022_9726_MOESM2_ESM.odt]

Copyleaks Plagiarism Report and Results

[https://copyleaks.com/dashboard/v1/businesses/report/x4tgyazf96ofbv1e/preview?key=us4ztgj7ht0n6gqb&suspectId=d1a0745f7c&viewMode=one-to-one&contentMode=html&sourcePage=1&suspectPage=1](https://copyleaks.com/dashboard/v1/businesses/report/x4tgyazf96ofbv1e/preview?key=us4ztgj7ht0n6gqb&suspectId=d1a0745f7c&viewMode=one-to-one&contentMode=html&sourcePage=1&suspectPage=1)
